# Health-related quality of life in adolescents and the retinal microvascular structure

**DOI:** 10.1038/s41598-018-21471-1

**Published:** 2018-02-15

**Authors:** Bamini Gopinath, Gerald Liew, George Burlutsky, Louise A. Baur, Paul Mitchell

**Affiliations:** 1 Centre for Vision Research, Department of Ophthalmology and Westmead Institute for Medical Research, University of Sydney, Sydney, NSW Australia; 2 University of Sydney Clinical School, The Children’s Hospital at Westmead, Sydney, NSW Australia; 3 School of Public Health, University of Sydney, Sydney, NSW Australia

## Abstract

We aimed to investigate whether health-related quality of life (HRQoL) is associated with retinal vascular caliber, an indicator of subclinical cardiovascular disease risk. 1600 students aged between 11–19 years (821 girls and 779 boys) were examined during 2009–2011. Retinal vessel caliber was measured from digital retinal images. HRQoL was assessed by the Pediatric Quality of Life Inventory (PedsQL). In the overall cohort, each 1-unit increase in PedsQL total score and the psychosocial summary score was associated with ~0.05 μm narrowing in retinal arteriolar caliber (multivariable-adjusted p-value = 0.01). Participants in the lowest versus highest tertile of PedsQL total score, psychosocial summary, social and school item scores had significantly wider retinal arteriolar caliber: 161.7 μm versus 160.2 μm (p = 0.02); 161.6 μm versus 160.0 μm (p = 0.02); 161.6 μm versus 159.9 μm (p = 0.002); and 161.6 μm versus 159.9 μm (p = 0.01), respectively. Significant interactions (p < 0.05) were observed between gender and PedsQL total score with retinal arteriolar calibre. In boys, inverse associations were observed between PedsQL total score (p = 0.01), psychosocial summary (p = 0.01), and social scores (p = 0.01) and retinal arteriolar caliber. No significant associations were observed between PedsQL scores and retinal vessel caliber in girls. Diminished HRQoL in adolescents was independently associated with structural retinal microvascular changes.

## Introduction

Health-related quality of life (HRQoL) refers to the subset of quality of life directly related to an individual’s health^1^, which as defined by the World Health Organization includes physical, mental, and social well-being^2,3^. Recent studies have shown a significant influence of HRQoL on long-term outcomes. For instance, poor HRQoL has been shown to be a marker of morbidity and mortality in patients with coronary heart disease, even after controlling for traditional vascular risk factors^4–6^. Moreover, HRQoL has also been reported to be associated with several cardiovascular risk factors, including obesity^7,8^, diabetes^9^ and smoking^10^.

Microvascular abnormality may underlie the association between subsets of HRQoL, particularly mental wellbeing, and vascular disease^11–13^. The retina offers a readily accessible site to non-invasively evaluate the microcirculation and is a recognized marker of early subclinical vascular disease^14^. Reliable methods of quantifying retinal microvascular changes from retinal photographs have been developed^15^, and subtle changes in the retinal vasculature may mirror pre-clinical structural changes in both the cerebral^16^ and coronary microcirculations^17^, and thus, could have prognostic information useful for predicting clinical cardiovascular events^18^. Further, structural retinal microvascular changes have shown to be associated with cardiovascular disease risk factors, including obesity and hypertension^19,20^.

While studies in adults and adolescents have shown that depression and anxiety symptoms are associated with measurable signs in the retinal microvasculature^11–13^; there have been no population-based studies that have examined the associations between overall HRQoL and retinal vessel caliber in either adults or children. Therefore, we aimed to assess the cross-sectional relationship between HRQoL (exposure) and retinal vessel caliber (study outcome) using a large cohort of adolescents. We hypothesize that lower HRQol scores is independently associated with modest structural retinal microvascular changes in adolescents. Hence, the epidemiological data from this study will help elucidate whether microvasculature structural alterations might be involved in the early mechanisms leading to cardiovascular disease and impaired HRQoL in later life.

## Participants and Methods

### Study Population

The Sydney Childhood Eye Study is a population-based survey of eye conditions in school children living within the Sydney Metropolitan Area, Australia. It was approved by the Human Research Ethics Committee, University of Sydney, the Department of Education and Training, and Catholic Education Office, New South Wales, Australia^21^. We obtained informed written consent from at least one parent of each child, as well as verbal assent from each child before the examinations. Study methods have been previously described^21^. All methods in this study were performed in accordance with the relevant guidelines and regulations. Briefly, Year 1 students from a stratified random cluster sample of 34 primary schools across Sydney (6–7 years of age) were eligible to participate. Data for the 6-year-old cohort (n = 1740) were collected during 2003–2004; and of these 1125 (64.7%) were followed up 5 years later i.e. aged 11–12 years. Also, students in Year 7 (12–13 years of age) in a stratified random cluster sample of 21 high schools across Sydney were eligible to participate. Data for this 12-year-old cohort (n = 2353) were collected during 2004–2005. Of the baseline participants 12 years of age, 1217 (51.7%) were resurveyed 5 years later (during 2009–2011), and includes children 16–19 years of age. Additional participants (n = 475) aged 17–18 years were recruited when we visited the same schools as in the baseline survey. Therefore, the total number of participants surveyed at the 5-year follow-up was 2817; comprising 1125 participants aged 11–12 and 1692 participants aged 16–19 years. Information on retinal vessel caliber was collected at baseline and follow-up surveys; however, health-related HRQoL was collected only at the 5-year examination. Therefore, the current study reports on cross-sectional data obtained from the 5-year survey only.

### Assessment of HRQoL

The Pediatric Quality of Life Inventory (PedsQL) 4.0 was used to assess HRQoL in preadolescents and adolescents and was only administered at the 5-year follow-up. The PedsQL is a validated 23-item questionnaire for children aged 2 to 18 years^22^, which takes students approximately 5–7 minutes to complete. The self-reported version of PedsQL was used in this study. Students reported how much of a problem they are experiencing in each of the PedsQL items in the past one month and mean scores were calculated based on a 5-point response scale for each item: (i) 0 - if it is never a problem; (ii) 1 - if it is almost never a problem; (iii) 2 - if it is sometimes a problem; (iv) 3 – if it is often a problem; and (v) 4 – if it is almost always a problem. Mean scores are then transformed to a 0 to 100 scale with a higher score representing better HRQoL. The PedQL yields 3 summary scores: a total scale score, a physical health summary score and a psychosocial health summary score. There are 4 sub-scale scores: physical functioning, emotional functioning, social functioning and school functioning. The total score is comprised of the average of all items in the questionnaire. The psychosocial summary is an average of the items in the emotional, social and school functioning scales. The physical health summary score comprises the average of items in the physical functioning scale^22^.

### Retinal Photography and Analysis

Children had dilated, digital photographs taken of the optic disk and macula of both eyes using a Canon 60UVID10 fundus camera (Canon Inc., Tokyo, Japan). For this study, retinal vascular caliber measurements for the right eye of each child were used. Left eye measurements were used when, occasionally, the photographs of the right eye were not gradable. One grader, masked to participant identity and characteristics, measured retinal vessel width using a computer-assisted program (Retinal Analysis, University of Wisconsin) with high reproducibility^15,23^. The six largest arteriolar and venular diameters measured for each eye, were then summarized as central retinal arteriolar equivalent (CRAE) or central retinal venular equivalent (CRVE), respectively, using a formula developed by Knudtson-Hubbard^24^.

### Assessment of Covariates

Parents were asked to complete a comprehensive 193-item questionnaire (at baseline and follow-up), from which socio-demographic information including ethnicity, and highest level of parental education was collected. Data were collected during a pre-organized visit to each school. Anthropometric measures were obtained by a trained medical officer. Height was measured to the nearest 0.1 cm with shoes off using a freestanding SECA height rod (Model 220, Hamburg, Germany). Weight in kilograms was measured to the nearest 0.1 kg using a standard portable weighing machine, after removing any heavy clothing. BMI was calculated as weight divided by height squared (kg/m^2^). After 5 minutes resting, blood pressure (BP) was measured in a seated position using an automated sphygmomanometer (HEM 907; Omron Healthcare Inc) with appropriate cuff size. Three separate BP measurements were taken, and averaged for analysis. Mean arterial BP (MABP) was calculated as one-third of the systolic plus two thirds of the diastolic BP.

A Canon autorefractor (Model RK-F1, Canon Inc., New York, U.S.A.) was used to perform cycloplegic autorefraction and keratometry. Children also had a comprehensive eye examination, which included mydriatic digital retinal photography. Axial length was measured before cycloplegia with an optical biometer (IOLMaster; Carl Zeiss Meditec, Oberkochen, Germany), using dual-beam partial coherence interferometry^25^. The average of 5 measurements was used for analysis.

### Statistical Analyses

Statistical analyses were performed using SAS (v9.2, SAS Institute, NC) including t-tests, χ^2^-tests and linear regression. Linear regression models were constructed to examine possible cross-sectional associations between HRQoL (independent variable) with retinal vascular caliber (dependent variable). PedsQL item scores were included in the analyses as continuous (each 1-unit increase) or as categorical variables (tertiles). Covariates that were previously found to be significantly associated with retinal vessel caliber^26,27^ were included in the final multivariable models i.e. age, sex, ethnicity, BMI, mean arterial BP, and axial length. Subsequently, analysis of covariance was used to calculate differences in mean retinal vascular caliber adjusted for age, sex, ethnicity, BMI, mean arterial BP, and axial length. After multivariable adjustment, linear regression analyses indicated interactions between sex and the associations of PedsQL total score (*P*_interaction_ = 0.02); psychosocial summary (*P*_interaction_ = 0.02); social item (*P*_intera ction_ = 0.001); and school item (*P*_interaction_ = 0.02) with retinal arteriolar caliber. Therefore, analyses of PedsQL and retinal vascular caliber were also stratified by sex.

## Results

Of the 2817 participants re-examined 5 years later, 1600 participants had complete information on retinal vessel caliber and PedsQL scores. Participants compared to non-participants were likely to be older and Caucasian, and to have higher BMI and systolic BP (Table 1). Table 2 shows the study characteristics of the 1600 participants stratified by gender. Significant differences were observed between boys and girls in regards to ethnic distribution, systolic and diastolic BP, axial length and in PedsQL total score, physical summary, emotional and social domain scores. For instance, boys compared to girls were more likely to be Caucasian, have higher systolic BP, axial length and PedsQL total scores (Table 2).

**Table 1:** Study characteristics of participants compared to non-participants.

Characteristics	Non-participants^a^ (n = 1217)	Participants (n = 1600)	*P*-value
Age, years, mean (SD)	14.3 (1.7)	16.3 (1.8)	<0.0001
Male, n (%)	625 (51.3)	779 (48.7)	0.17
Ethnicity, n (%)			
Caucasian	583 (46.3)	1020 (63.8)	<0.0001
East Asian	388 (30.8)	229 (14.3)	
South Asian	53 (4.2)	77 (4.8)	
Middle Eastern	89 (7.1)	116 (7.3)	
Other	146 (11.6)	158 (9.9)	
Body mass index, kg/m^2^, mean (SD)	20.8 (4.2)	22.2 (4.4)	<0.0001
Systolic blood pressure, mm Hg, mean (SD)	117.1 (13.2)	119.0 (13.3)	<0.0001
Diastolic blood pressure, mm Hg, mean (SD)	67.8 (9.5)	68.4 (9.6)	0.14
Axial length, mm, mean (SD)^1^	23.6 (1.0)	23.6 (0.9)	0.02
PedsQL			
Total score	79.7 (12.7)	80.8 (12.3)	0.15
Physical summary	88.6 (14.4)	89.9 (13.3)	0.11
Psychosocial summary	75.3 (14.6)	76.2 (14.6)	0.28
Emotional	72.5 (19.1)	74.6 (19.2)	0.08
Social	88.7 (15.5)	88.9 (14.9)	0.80
School	65.7 (22.4)	65.8 (22.1)	0.93

Data presented are mean (SD) or proportions.

^a^Non-participants are those who were excluded from analysis because they had incomplete information on retinal vessel caliber and/or PedsQL scores.

**Table 2:** Study characteristics of participants (n = 1600), stratified by gender.

Characteristics	Girls (n = 821)	Boys (n = 779)	*P*-value
Age, years, mean (SD)	16.3 (1.7)	16.2 (1.8)	0.44
Ethnicity, n (%)			
Caucasian	491 (59.8)	530 (68.0)	0.003
East Asian	140 (17.1)	89 (11.4)	
South Asian	43 (5.2)	34 (4.4)	
Middle Eastern	57 (6.9)	58 (7.5)	
Other	90 (11.0)	68 (8.7)	
Body mass index, kg/m^2^, mean (SD)	22.2 (4.5)	22.2 (4.2)	0.88
Systolic blood pressure, mm Hg, mean (SD)	114.1 (11.2)	124.3 (13.4)	<0.0001
Diastolic blood pressure, mm Hg, mean (SD)	69.4 (9.4)	67.3 (9.8)	<0.0001
Axial length, mm, mean (SD)^1^	23.4 (0.9)	23.9 (0.9)	<0.0001
PedsQL			
Total score	79.7 (13.1)	81.9 (11.4)	0.0001
Physical summary	87.5 (14.6)	92.0 (12.0)	<0.0001
Psychosocial summary	75.8 (15.1)	76.7 (14.0)	0.25
Emotional	71.8 (20.4)	77.4 (17.4)	<0.0001
Social	90.1 (13.7)	87.6 (15.9)	0.001
School	66.9 (22.4)	64.8 (21.6)	0.06

Data presented are mean (SD) or proportions.

In the overall cohort, each 1-unit increase in PedsQL total score and the psychosocial summary score were significantly associated with ~0.05 μm narrowing in retinal arteriolar caliber (Table 3). After multivariable adjustment for age, sex, ethnicity, body mass index, mean arterial blood pressure, and axial length, a significant narrowing of retinal arteriolar caliber was also observed with each 1-unit increase in the PedsQL emotional (p = 0.04), social (p = 0.01) and school subscale scores (p = 0.04). Each 1-unit increase in the PedsQL social item score was significantly associated with ~0.07 μm widening of retinal venular caliber, no other significant associations were observed with retinal venules (Table 3). In boys, similar inverse associations were observed between PedsQL total score (p = 0.01), psychosocial summary (p = 0.01), and social scores (p = 0.01) and retinal arteriolar caliber after multivariable adjustment (Table 4). Also, each 1-unit increase in the PedsQL social item score was associated with ~0.09 μm widening of retinal venular caliber (multivariable-adjusted p-value = 0.01). No significant associations were observed between PedsQL item scores and retinal vascular caliber measures in girls (data not shown).

**Table 3:** Associations between PedsQL scores and retinal vessel caliber in adolescents.

PedsQL scores (each 1-unit increase)	Retinal vascular caliber, mean (SE)^a^			
	Arteriolar caliber, μm	*p*-value	Venular caliber, μm	*p*-value
Total score	−0.05 (0.02)	0.01	0.03 (0.03)	0.40
Physical summary	−0.01 (0.02)	0.54	−0.008 (0.03)	0.78
Psychosocial summary	−0.05 (0.02)	0.004	0.03 (0.03)	0.24
Emotional	−0.03 (0.01)	0.04	0.001 (0.02)	0.64
Social	−0.05 (0.02)	0.01	0.07 (0.03)	0.01
School	−0.02 (0.02)	0.04	0.001 (0.02)	0.96

^a^Adjusted for age, sex, ethnicity, body mass index, mean arterial blood pressure, axial length.

**Table 4:** Associations between PedsQL scores and retinal vessel caliber in boys.

PedsQL scores (each 1-unit increase)	Retinal vascular caliber, mean (SE)^a^			
	Arteriolar caliber, μm	*p*-value	Venular caliber, μm	*p*-value
Total score	−0.07 (0.03)	0.01	0.06 (0.04)	0.24
Physical summary	−0.02 (0.03)	0.54	−0.02 (0.05)	0.67
Psychosocial summary	−0.07 (0.03)	0.01	0.06 (0.04)	0.11
Emotional	−0.04 (0.02)	0.06	0.02 (0.03)	0.52
Social	−0.06 (0.02)	0.01	0.09 (0.03)	0.01
School	−0.03 (0.02)	0.07	0.01 (0.03)	0.56

^a^Adjusted for age, ethnicity, body mass index, mean arterial blood pressure, axial length.

Table 5 shows that in the overall cohort, those in the lowest tertile compared to those in the highest tertile of PedsQL total score, psychosocial summary, social and school item scores had significantly wider retinal arteriolar caliber: 161.7μm versus 160.2 μm (p = 0.02); 161.6 μm versus 160.0 μm (p = 0.02); 161.6 μm versus 159.9 μm (p = 0.002); and 161.6 μm versus 159.9 μm (p = 0.01), respectively. Similar significant associations were observed in boys after multivariable adjustment (Table 5). No significant associations were observed between tertiles of PedsQL item scores and retinal arteriolar caliber in girls (data not shown).

**Table 5:** Associations between tertiles of PedsQL scores and retinal vessel caliber in adolescents.

PedsQL scores	Overall	Boys
	Arteriolar caliber, μm	Arteriolar caliber, μm
Total score		
1^st^ tertile (≤75.8)	**161.7 (160.6–162.9)**	**161.2 (159.6–162.9)**
2^nd^ tertile (76.7–86.7)	160.1 (158.9–161.2)	159.5 (158.1–160.9)
3^rd^ tertile (≥87.1)	160.2 (159.1–161.4)	159.1 (157.5–160.6)
*P* for trend	0.02	0.02
Physical summary		
1^st^ tertile (≤85.0)	160.8 (159.7–162.0)	160.1 (158.4–161.8)
2^nd^ tertile (90.0–95.0)	161.1 (159.8–162.4)	160.4 (158.7–162.1)
3^rd^ tertile (≥100.0)	160.3 (159.3–161.4)	159.5 (158.1–160.8)
*P* for trend	0.51	0.39
Psychosocial summary		
1^st^ tertile (≤67.5)	**161.6 (160.4–162.8)**	**160.8 (159.2–162.5)**
2^nd^ tertile (70.0–82.5)	160.5 (159.4–161.6)	159.9 (158.5–161.3)
3^rd^ tertile (≥83.8)	160.0 (158.9–161.2)	158.9 (157.3–160.4)
*P* for trend	0.02	0.03
Emotional		
1^st^ tertile (≤62.5)	161.1 (159.9–162.2)	160.3(158.6–161.9)
2^nd^ tertile (68.8–81.3)	160.7 (159.6–161.9)	160.3 (158.8–161.8)
3^rd^ tertile (≥87.5)	160.1 (159.0–161.2)	159.1 (157.6–160.5)
*P* for trend	0.13	0.16
Social		
1^st^ tertile (≤83.3)	**161.6 (160.5–162.8)**	**160.8 (159.4–162.2)**
2^nd^ tertile (87.5–91.7)	161.4 (160.0–163.0)	160.4 (158.4–162.5)
3^rd^ tertile (≥100)	159.9 (158.8–160.9)	158.9 (157.5–160.2)
*P* for trend	0.002	0.02
School		
1^st^ tertile (≤50.0)	**161.6 (160.4–162.8)**	**160.7 (159.2–162.2)**
2^nd^ tertile (58.3–75.0)	160.4 (159.4–161.5)	159.7 (158.3–161.2)
3^rd^ tertile (≥83.3)	159.9 (158.7–161.1)	158.7 (157.1–160.4)
*P* for trend	0.01	0.04

Bolded values indicate significant (p < 0.05) estimates in comparison with the highest or 3^rd^ tertile.

^a^Adjusted for age, sex, ethnicity, body mass index, mean arterial blood pressure, axial length.

Participants in the lowest tertile compared to the highest tertile of social scores had significantly narrower retinal venules: 235.1 versus 236.9 μm; multivariable-adjusted p = 0.03. Similarly, boys in the lowest tertile compared to the highest tertile of social scores had significantly narrower retinal venules: 234.7 versus 238.1 μm; multivariable-adjusted p = 0.04. No significant associations between PedsQL scores and retinal venular caliber were observed in girls (data not shown).

## Discussion

This is the first epidemiological study to demonstrate an independent association between HRQoL and structural retinal microvascular changes in a young population. Specifically, we show a modest cross-sectional association between lower HRQoL scores with wider retinal arteriolar caliber and narrower retinal venular caliber in a cohort of adolescents. In particular, the psychosocial aspects of HRQoL in adolescents showed a significant association with retinal vessel caliber changes. Further, observed associations were more marked in boys compared to girls.

There are few other cohort studies with which to compare our findings with. The most relevant studies are those showing significant associations between depression and anxiety and wider retinal arteriolar caliber in adolescents^11^ and adults^12,13^. This is in agreement with our data which showed that lower PedsQL psychosocial health summary scores (i.e. average of the items in the emotional, social and school functioning scales) were associated with retinal arteriolar dilatation. The specific pathophysiological mechanisms underlying wider retinal arteriolar caliber are unclear, but one speculated pathway is endothelial dysfunction (impairment of nitric oxide-mediated vasodilation)^11^. Wider retinal arteriolar caliber is a sign of impaired autoregulation^28^ and is associated with reduced flicker-induced vasodilatation^11,29^. Reduced endothelial function was associated with worse HRQoL in a prior study involving adolescents and young adults^30^, and endothelial dysfunction has also been observed in individuals with depression and anxiety^31,32^. In the present study, however, we were not able to assess endothelial function; hence, we are not able to confirm whether endothelial dysfunction is a potential mechanism underlying the link between HRQoL and retinal arteriolar dilatation. Additional studies would be needed to answer this question.

Another potential underlying mechanism is inflammation. There is research evidence to suggest that inflammatory biomarkers are meaningful correlates of HRQoL and robust inverse associations have been demonstrated between C-reactive protein (an inflammatory marker) and HRQoL^33^. Moreover, several studies reported that psychosocial factors were predictors of chronic inflammation and poor health^34^. The influence of psychological well-being on C-reactive protein may be supported by studies that reported an association between psychological stress and the dysregulation of the hypothalamic-pituitary-adrenal axis involved in inflammation^35^. Given that changes to retinal vascular calibers are related to systemic inflammation^36^, this could be a possible pathway by which HRQoL is independently associated with retinal microvascular signs in adolescents. Future prospective studies with adequate study power are warranted to focus on mechanisms to help explain these structural changes in retinal vessels in relation to poorer HRQoL.

We observed a sex-specific difference in the associations between PedsQL scores and retinal microvascular signs. These data align with the existing research evidence showing differences in the association of vascular disease risk factors with HRQoL between men and women e.g., for obesity^7,8^ and smoking^37^. Furthermore, *Gijsberts et al*.^38^ showed significant interactions of gender with diabetes and history of cardiovascular disease. Specifically, the association of these vascular risk factors with lower HRQoL was stronger in men than in women. The authors speculated that HRQoL in girls is not so much determined by CVD risk factors, a history of CVD or other general patient characteristics but more by other factors such as hormonal status and psychosocial factors that were not measured in their study^38^. We hypothesize that this is also likely to be applicable in our cohort study. Finally, our findings support data from a longitudinal study which found that the associations between mental stress with adverse changes to the vascular function and vascular response were more pronounced in boys rather than girls^39^. Together these epidemiological data, suggest that boys are likely to have changes in vascular health earlier than age-matched girls^39^.

Strengths of this study include its random cluster sample of a relatively large number of representative schoolchildren; satisfactory response rate; use of standardized retinal vessel caliber measurement protocols and a validated pediatric HRQoL instrument. Moreover, these young children were largely free of known systemic cardiovascular diseases, and thus, our findings are not likely to be subject to confounding effects CVD risk factors^26^. A study limitation is the cross-sectional design as PedsQL was not administered at the baseline survey, which does not permit causal inference from the observed associations. Second, we did not collect blood samples in our study; therefore, there is no data on biomarkers of chronic disease e.g. blood lipid and glucose levels, and measures of inflammatory markers. Third, given that participants versus non-participants differed significantly in e.g. age, ethnicity and BMI, we cannot disregard the potential for selection bias influencing observed associations. Finally, while we adjusted for a number of important confounders, we cannot discount the possibility that other unmeasured factors such as parental well-being and societal factors could have influenced HRQoL in adolescents.

In summary, our community-based study has made an original contribution by showing an independent association between poorer HRQoL scores and retinal microvascular abnormalities in adolescents. As these subtle structural retinal microvascular changes have shown to be markers of future vascular disease risk, the presence of this risk factor in schoolchildren could support the need for clinicians and researchers to incorporate assessments of HRQoL when evaluating the cardiovascular health of adolescents.
